# Intense focused ultrasound stimulation can safely stimulate inflamed subcutaneous tissue and assess allodynia

**DOI:** 10.1186/2050-5736-2-8

**Published:** 2014-02-25

**Authors:** Abbi M McClintic, Josephine B Garcia, Michael Gofeld, Michel Kliot, John C Kucewicz, John D Loeser, Kristin D Pederson, Rachel E Sparks, Gregory W Terman, Rowen E Tych, Pierre D Mourad

**Affiliations:** 1 Department of Neurological Surgery, University of Washington, Box 356470, Seattle, WA, USA; 2 Department of Anesthesiology and Pain Management, University of Washington, Seattle, WA, USA; 3 Applied Physics Laboratory, University of Washington, Seattle, WA, USA; 4 Department of Bioengineering, University of Washington, Seattle, WA, USA

**Keywords:** Focused ultrasound, Inflammatory pain, Allodynia

## Abstract

**Background:**

Potential peripheral sources of deep pain can require invasive evocative tests for their assessment. Here we perform research whose ultimate goal is development of a non-invasive evocative test for deep painful tissue.

**Methods:**

We used a rat model of inflammation to show that intense focused ultrasound (iFU) differentially stimulates inflamed versus control tissue and can identify allodynia. To do so we applied iFU to inflamed and normal tissue below the skin of rats’ hind paws and measured the amount of ultrasound necessary to induce paw withdrawal.

**Results:**

iFU of sufficient strength (spatial and temporal average intensities ranged from 100–350 W/cm^2^) caused the rat to withdraw its inflamed paw, while the same iFU applied to the contralateral paw failed to induce withdrawal, with sensitivity and specificity generally greater than 90%. iFU stimulation of normal tissue required twice the amount of ultrasound to generate a withdrawal than did inflamed tissue, thereby assessing allodynia. Finally, we verified in a preliminary way the safety of iFU stimulation with acute histological studies coupled with mathematical simulations.

**Conclusions:**

Given that there exist systems to guide iFU deep to the skin, image-guided iFU may one day allow assessment of patient’s deep, peripheral pain generators.

## Background

A non-invasive diagnostic test that could reliably assess subcutaneous, peripheral contributors to a patient’s pain would be extremely helpful for diagnosing and treating patients with pain of unknown origin. Current diagnostic tests have limited ability to locate and assess the contribution of subcutaneous tissue to a patient’s experience of pain. For example, in up to 85% of patients with back pain, imaging studies and physical examination cannot pinpoint anatomic structures responsible for generating the pain because they appear normal or because multiple anatomic changes appear in images [1], all complicated by the likely presence of central sensitization [2]. Evocative tests such as discography address the non-specificity of imaging for locating pain by correlating the patient’s report of pain with increased intradiscal pressure generated by injection of fluid within the disc. Unfortunately, this approach is invasive and uncomfortable for the patient. Moreover it tests only a subset of candidate peripheral pain generators, hence has relative low sensitivity and specificity.

As reviewed by Woolf [2], contributions to a patient’s experience of pain caused by central sensitization include pain from normally non-painful stimulation (allodynia), increased pain response to normally painful stimulation (hyperalgesia), enhanced temporal summation, (where a patient’s experiences increased pain during the application of a series identical but rapidly applied stimuli) and the possibility that subclinical but irritating signals from deep peripheral tissue chronically stimulate and thereby maintain central sensitization. With this insight, current clinical practice for chronically painful diseases has begun to assess these characteristics of pain in patients compared to healthy controls. For example, clinicians use pulses of known amounts of heat, electricity or pressure applied to the skin of potential fibromyalgia patients that generally produces more intense sensations or even pain, of duration generally far beyond that of the stimulation itself, when compared to its application to healthy test subjects [3-6]. Though comparable results have been produced by focal application of pressure to the skin in a manner shown to activate subcutaneous sensory fibers after application of a topical anesthetic [4], this method of assessing the extent of pain cannot isolate potential sources of subcutaneous peripheral irritation since its stimulation involves all subcutaneous tissues.

We believe that the clinical management of pain associated with subcutaneous tissue could benefit from a non-invasive, targeted and quantifiable evocative test for deep-tissue pain. Intense focused ultrasound could form the basis of this test. Specialized ultrasound devices can focus quantifiable amounts of ultrasonic energy within a roughly cylindrically shaped volume of tissue on the order of tens of microliters that lies subcutaneous within the body [7,8] sufficient to cause rapid, transient, and localized increase in tissue temperature. Such devices can also cause local tissue displacement, hence localized shear forces within tissue [7,8]. In sufficient quantities (generally greater than 1000 W/cm^2 with application times greater than a second), the heat, shear, and/or cavitation – the formation and activation of mechanically active bubbles by ultrasound [8] – can destroy tissue [9-11]. Such ultrasound, of high intensity and designed to alter tissue properties generally for therapeutic purposes, is generally known as high intensity focused ultrasound – HIFU. Using the same HIFU technology but with shorter durations and lower intensities, intense focused ultrasound (iFU) can safely induce discernible sensations in human subjects [12,13].

Given this insight, Gavrilov [12,14,15], Wright [16], and their colleagues suggested that iFU applied to tissue associated with a potential neuropathic injury would elicit abnormal sensations discernibly different from those elicited by iFU applied to normal tissue. We have successfully tested their hypothesis directly [17,18]. We have also observed that diurnally inflamed tissue varies in its response to individual and short pulses of iFU [19]. In addition we studied the effect of multiple and rapidly delivered short pulses of iFU applied to inflamed tissue compared to contralateral tissue [20].

Here we sought to test the hypothesis that iFU can differentiate inflamed, diffusely painful subcutaneous tissue from contralateral subcutaneous tissue. We also assessed whether or not iFU could diagnose allodynia by using separate cohorts of animals to compare iFU threshold values of inflamed tissue and *normal* tissue. Finally we present histological results consistent with the safety of this procedure and calculations that assess the likelihood of damage caused by our iFU protocols.

## Materials and methods

All animal procedures were approved by the Institutional Animal Care and Use Committees of both the University of Washington and the Veterans Administration of Puget Sound.

### Animal model of peripheral inflammatory pain

Adult male Fischer rats (approximately 180 g, Charles River) were anesthetized with a 5% Isoflurane (Pitman-Moore, Mundelein, IL) and oxygen mixture via nose cone for induction. The concentration was decreased to 2% Isoflurane for maintenance of the anesthetic. Inflammation was induced using methods adapted from Nagakura et al. [21]: 0.2 ml of Complete Freund’s Adjuvant (CFA, Sigma Aldrich) was injected subcutaneously over 45 seconds into the plantar surface of the right hind paw at the base of the toes using a 25 g 5/8” needle. This produced significant inflammation throughout the right hind paw — from skin to periosteum — with maximum sensitivity observed 5–7 days after injection [21]. A separate group of rats did not receive the CFA injection, serving as normal controls to allow assessment of allodynia by comparison of iFU stimulation values for inflamed versus normal paws.

### Ultrasound devices and acoustic protocols

For this study we used two ultrasound devices, which we first summarize here. We used a device optimized for subcutaneous stimulation for our behavioral studies, which assessed allodynia. We used a laser-guided device for the safety studies where we applied iFU to very specific, subcutaneous tissue, facilitated by use of lasers to identify the focus in tandem with a micropositioning device. This allowed us to assay in a preliminary way the margin of safety of subcutaneously delivered iFU for stimulation by identifying sufficient iFU to cause acute, observable tissue damage. The center of the focus (peak pressure value) occurred within 4–7 mm beyond the tip of both devices.

The transducer used for our behavioral studies generated ultrasound using the inner element (22.6 mm inner diameter, 48.5 mm outer diameter) of a two-element, 1.1 MHz transducer (H-105 S/N-01 Sonic Concepts, Inc., Bothell, WA). Ultrasound was beamed to the tissue of interest through a plastic removable cone mounted on the transducer and filled with degassed water. The laser-guided device for our safety study consisted of a 2 MHz single-element (35 mm diameter) focused transducer (SU-101 - http://www.sonicconcepts.com/images/pdf/su-101_datasheet.pdf, Sonic Concepts, Inc., Bothell, WA) on which a plastic removable cone was mounted on the transducer and filled with degassed water facilitate ultrasound propagation as above. Mounted permanently on the sides of this cone were 2 lasers (Digi-Key, Thief River Falls, MN) pointed at the center of the ultrasound focus, useful here for directing the focus of our device to the appropriate exposed tissue for our safety study.

Acoustic field maps were generated for both transducers. The acoustic pressure was simulated using custom MATLAB (The Mathworks, Inc., Natick, MA) software written in-house for modeling arbitrary transducer geometries assuming linear acoustics [22,23]. With those simulations we calculated the full width half maximum (FWHM) pressure, the intensity, and the fractional ultrasound power within the FWHM as well as the area within the FWHM for each transducer [23,24]. Table 1 provides the geometric specifications of our transducers.

**Table 1:** Geometric parameters of our ultrasound devices

**Device**	**Transaxial length at half maximum pressure**	**Axial length at half maximum pressure**	**% of energy within length of beam at half maximum pressure**
**Behavioral**	0.6 mm	7.5 mm	44.7%
**Laser-guided**	0.8 mm	8.45 mm	70%

The transducers were driven by an amplifier (A150 RF Power Amplifier, ENI, Chesnut Ridge, NY) controlled by two function generators (33120A, Hewlett Packard/Agilent, Palo Alto, CA). The first generator gated the pulse to a specific duration. The second, in series with the first, modified the acoustic output and ensured that the pulse was emitted at a specific frequency. The amplifier increased the signal from the function generators and sent it to the device. An oscilloscope (Wave Runner LT 322, LeCroy, Chesnut Ridge, NY) measured the duration of the pulse, its carrier frequency and the voltage delivered to the iFU device by the amplifier during each experiment.

The acoustic output of each transducer as a function of input voltage was measured using a radiation force balance [24]. Our intensity measures are reported in terms of spatial average temporal average intensity, I_SATA_, which we define as the total acoustic power of the transducer at a given input voltage times the fractional power within the FWHM divided by the area enclosed by the FWHM [23,24].

We also applied a pulse of 0.375 seconds using the behavioral device to the paws of both normal rats and those with inflamed paws in order to demonstrate that iFU can be used to assess allodynia in subcutaneous tissue.

### Behavioral data collection - Hargreaves and iFU application to rats

We performed our studies starting five days after CFA injection. Groups of three rats underwent a set of Hargreaves heat pain paw withdrawal tests [25], followed by application of iFU and then another set of Hargreaves tests, all during the daytime of a single day, with each test separated from the next by an hour of waiting time. Rats were placed in separate compartments in a Plexiglas cage with a glass floor, and an intense light was applied serially to each rear paw of each rat through the floor via a focused lamp. When the rat withdrew its paw the lamp was turned off and the time to withdrawal was recorded. The lamp automatically turned off if the rat did not withdraw its paw after 20 seconds. All data was recorded in seconds. We repeated this test five times for each rat.

**Figure 1:**
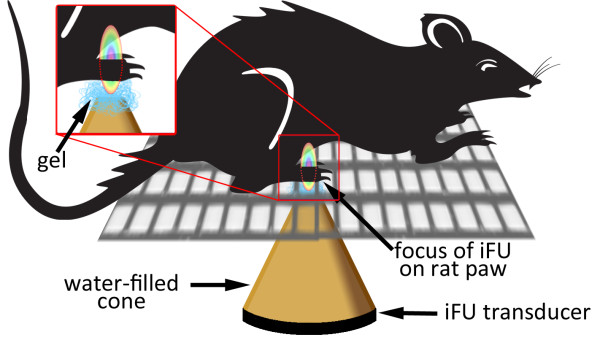
**Diagram of our experimental setup for determination of iFU withdrawal threshold.** A rat is placed on top of a mesh grid, allowing us to deliver iFU through the floor to the plantar aspect of the rat’s paws. Ultrasound gel is used to ensure adequate coupling of the device.

After heat lamp testing, we habituated sets of three rats to their free-ranging presence within individual cages containing three separate enclosures, each with a mesh bottom (rather than plexiglass bottom) whose individual holes were large enough to allow the researcher to pass through the distal tip of the ultrasound device to the bottom of the rat’s feet (Figure 1). Within this experimental setup we habituated the rats to a light touch of the iFU transducer and acoustic gel (Aquasonic 100 Ultrasound Transmission gel, Parker Laboratories Inc.) to the plantar aspect of their paws such that the rat would not move its paw despite the touch. This light touch plus use of a small volume of ultrasound gel placed between the transducer and the rat’s paw ensured adequate acoustic coupling between the two. Habituating the rats to the device took approximately 5 minutes per set of three rats, as indicated by an absence of reaction to the touch of the transducer and a lessening of exploratory movements.

**Figure 2:**
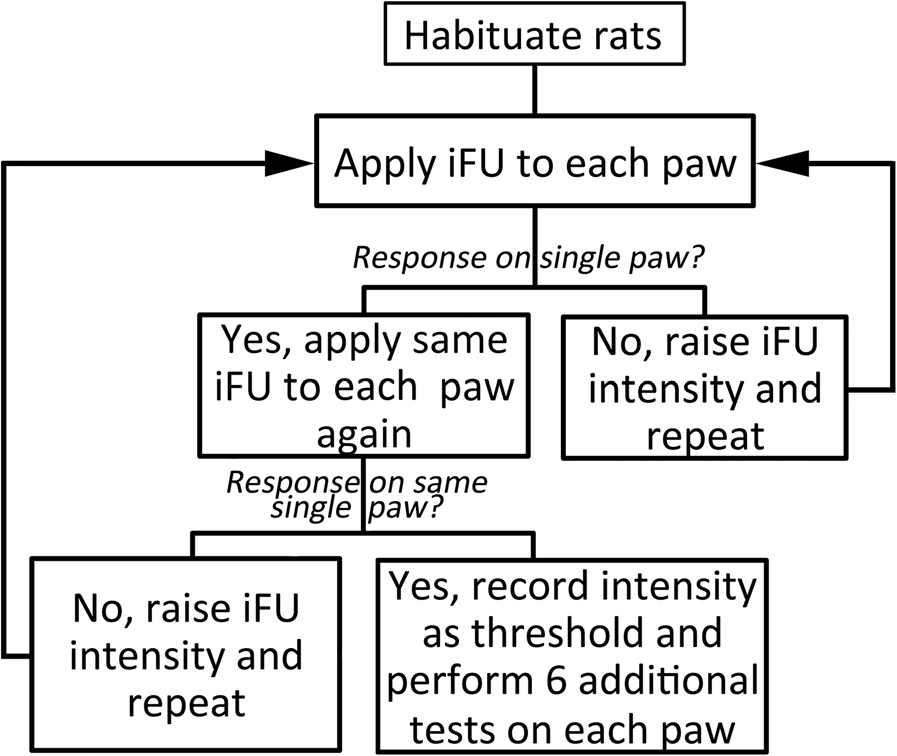
Flowchart of the iFU threshold determination process.

After habituation we again placed the proximal surface of the device up through the holes within the bottom of the mesh cage until that proximal surface touched the bottom of one of the rat’s hind paws, again using ultrasound gel to ensure adequate coupling. (Note that if during the iFU test procedure a rat began to withdraw its paw in response to contact with the device plus gel but without iFU application, it was re-habituated to the touch of the device plus gel before re-starting iFU threshold testing). This time we applied a single pulse to the plantar surface of the paw, seeking to observe an immediate and rapid withdrawal of the stimulated hind paw. We then applied iFU to the other hind paw in each of the three rats after a minimum of 30 seconds. The order of paw testing was randomized based on the rat’s position in the cage. In the absence of a hind paw withdrawal response the intensity of ultrasound was increased in increments beginning at 30% and tapering to 10% increase as intensity increased, starting with an initial acoustic intensity of approximately 50 W/cm^2^. Rats showing only one out of two withdrawal responses to a given level of iFU stimulation at a given power on a given paw were considered negative tests and the intensity was increased as described until we found the minimum iFU threshold intensity at a given duration that induced two consecutive withdrawal responses from a given paw. If rats withdrew both hind paws to a given iFU intensity the acoustic intensity was decreased and iFU was re-applied until only one paw withdrawal response was observed twice after each of two consecutive applications of iFU to that paw or we determined that we could not identify a single sensitive paw (see flowchart in Figure 2). In our experience this entire experimental procedure required one to two hours of effort for a set of three rats.

The intensity and dose of iFU that caused two consecutive withdrawals on the same paw for a given rat was defined as the iFU threshold value for that rat on that day. iFU was then applied six more times at that same value to facilitate calculations of sensitivity and specificity. We applied iFU to each rat on separate testing days. No rat received an entire iFU test more than once per day, with at least 1 day between each test. After testing, rats were returned to their cages and then returned to the animal housing facility.

### Data analysis

Data was entered into an Excel (Microsoft, Redmond, WA) spreadsheet where the intensity and dose of each acoustic protocol, latency times measured from the Hargreaves test, etc., were calculated and reported as aggregates in terms of an average +/- standard deviation. Differences in acoustic intensity or Hargreaves latency times between groups were evaluated by analyses of variance with Tukey’s tests for appropriate post-hoc comparisons (GB Stat; Dynamic Microsystems; Silver Springs, Maryland). Differences between two groups of data are reported as statistically significant if the p-value < 0.05.

We calculated the sensitivity and specificity of iFU application to the paws using data from both the iFU threshold value determination plus the six additional iFU applications of that same iFU threshold value to each of the rat’s rear paws. We define sensitivity as the number of withdrawal responses on the injured paw to the threshold intensity divided by the total applications to that paw at that intensity. Similarly, we defined the specificity as the number of applications of iFU at the threshold intensity to the uninjured paw that elicited no response divided by the total number of applications to that paw.

We used box plots to represent our results, where the line in the box gives the median value, the box encompasses the position of 75% of the values, the whiskers bound 95% of values and individual points represent individual values that extend beyond the 95% boundary.

### Safety data collection and calculations

#### ***iFU application to exposed thigh muscle***

In parallel to behavioral studies that anticipate application of this methodology to subcutaneous muscle, we collected acute safety data to demonstrate the margin of safety of stimulating skeletal muscle via iFU application. We elected to study muscle instead of the paw because histology experts suggested to us that the heterogeneous damage caused by the inflammation would likely obscure any damage caused by iFU. Therefore, we applied iFU directly to skeletal muscle at an intensity comparable to the threshold established in the behavioral studies, and systematically increased this value until we observed acute tissue damage deep to the surface of the exposed muscle using histological analysis. In our pilot studies we found that ultrasound comparable in intensity and dose to those used in our behavioral tests did not cause any acute damage (data not shown). We therefore selected for reporting here the histological effects of iFU at significantly larger intensity and acoustic dose values substantially greater than anything used in behavioral tests. In this way we identified the acute margin of safety of iFU stimulation.

Seven adult Sprague Dawley rats (approximately 200 g, Charles River) were deeply anesthetized with a 5% Isoflurane (Pitman-Moore, Mundelein, IL) and oxygen mixture via nose cone for induction and 2% Isoflurane for maintenance of the anesthesia. 0.1 mL of lidocaine was injected subcutaneously in the hind limb and a 2-cm long incision was subsequently made to expose the muscle at the mid-thigh level. The skin was retracted to the medial side and the biceps femoris muscle was exposed and dissected from surrounding fascia. Using 3–0 silk sutures to indicate the area of intended iFU application, calibrated lasers attached to the iFU device were used to place iFU application within the bounds of the suture markers. One iFU dose was applied per thigh.

Within an hour of iFU application, the animals were sacrificed with Beuthanasia (phenytoin, 390 mg/mL pentobarbital) and perfused via transcardiac injection with 4% paraformaldehyde in phosphate buffer. Then we dissected out a 1 cm^3^ tissue sample, taken from at and below the surface of the muscle at mid-thigh level from the ipsilateral side, as indicated by the sutures applied prior to application, along with comparable tissue from the contralateral hind limb. Tissue specimens were post-fixed in 4% paraformaldehyde in phosphate buffer, immersed in sucrose for cryoprotection, and embedded in diethylene glycol (OCT) for frozen sectioning. Serial sections (10 μm) were obtained using a cryostat at -20°C. These sections were stored in a freezer at -80°C and later stained using Hematoxylin & Eosin.

#### ***Theoretical estimation of the chance for damage due to iFU stimulation***

We wished to further assess the possibility that our iFU stimulation protocols could cause damage via tissue heating to the rats’ paws. To do this, we estimated the minimum intensity of iFU necessary to cause tissue damage based on theoretical concerns, given the parameters of our device and pulse duration. We then compared the estimated threshold for damage with the results of our behavioral studies. To calculate the estimated iFU damage threshold, we utilized the quantity ‘time for equivalent thermal dose’ (t_43_) as an intermediate variable.

The calculation to estimate the iFU damage threshold is described in the following steps:

(1) In MATLAB, we calculated the heat generated by our iFU device throughout a uniformly space grid in a 1 cm × 1 cm plane, normal to the axis of ultrasound propagation at the focus. We used a temporal step-size of 1 ms and total duration of 10s. The spatial structure of the intensity field for the transducer was scaled according to the spatial average temporal average (SATA) intensity under simulation. Tissue within the plane was assumed to be striated muscle. The tissue parameters used in the simulation are shown in Table 2[26]. We used the attenuation coefficient in our calculations, which includes losses due to absorption and scattering but is dominated by absorption [22].

(2) We used the Bioheat equation [27] to estimate the time variation in temperature generated by the heat generated by our device. We included thermal conduction, but not perfusion, for this calculation.

(3) We calculated the equivalent thermal dose (t_43_) using the time-varying temperature generated in step 2 in the equation below, where T is the temperature in °C and R is 0.5 for temperatures greater than 43°C and 0.25 for temperatures below 43°C [28]. The rat baseline body temperature is assumed to be 38°C [29]. Thermal damage is estimated to occur at a t_43_ of 240 minutes, or the equivalent of holding tissue at 43°C for 240 minutes [28].

$$t_{43}=\int_{t=0}^{\mathrm{final}}tR^{\left(43-T\left(t\right)\right)}\mathrm{dt}$$

(4) We followed steps 1–3 across a range of intensities, in order to determine at what intensity the t_43_ would reach the 240-minute damage threshold. This intensity was recorded as the minimum intensity at which thermal damage might occur.

**Table 2:** Tissue parameters for heating calculations

**Tissue type**	Specific heat (J/(g*C))	**Attenuation coefficient (Nepers/cm)**	**Density** **(g/cm**^**3**^**)**	Thermal conductivity (W/cm*C)
**Muscle**	3.72	0.15	1.07	0.0047

## Results

### Hargreaves results

Hargreaves testing on the rats did not alter the iFU threshold values. iFU did not alter the Hargreaves threshold values, whose average value equaled 4.1 seconds +/- 1.2 on the inflamed paw and 8.2 seconds +/- 1.7 on the normal paw.

### Assessment of allodynia for subcutaneous delivery of iFU

**Figure 3:**
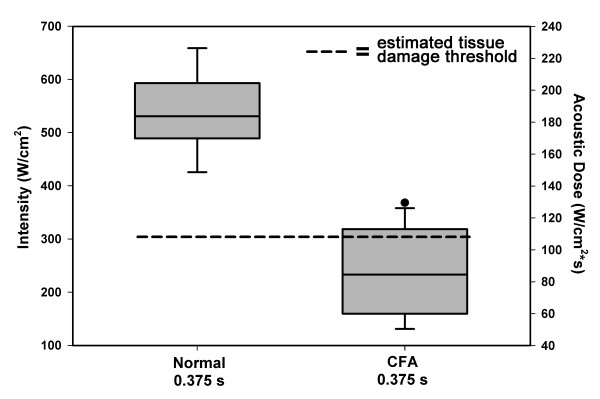
**Assessing allodynia with the deep focus iFU device.** Intensity (left axis) and dose (right axis) of iFU necessary to produce a reliable withdrawal from each of inflamed and normal paws, for the deep focus device. ‘CFA’ refers to rats whose rear paws were inflamed with Complete Freunds Adjuvant while ‘normal’ refers to rats with uninjured paws. The dashed line represents the value at which (or above) thermal damage may occur.

We performed twelve tests on nine rats with inflamed paws and nine tests on six rats with normal paws using a single acoustic pulse of 0.375 seconds in duration. We were able to identify iFU threshold values for all tests on rats with inflamed paws: the inflamed paw withdrew first 100% of the time. Also, we were able to identify iFU threshold values for all tests on rats with normal paws, with no difference in the number of right versus left paws responding first. The threshold intensity and dose values were significantly higher (p < 0.0001) for the control rats with normal paws versus the rats with inflamed paws, by an average multiplicative factor of two (Figure 3).

### Sensitivity and specificity of iFU stimulation

As discussed in the methods section we used the initial two applications of iFU to determine the threshold iFU value. We combined those data points with the six additional applications of iFU at the iFU threshold value to calculate values of sensitivity and specificity. The sensitivity for the inflamed paws was 89.6% +/- 10.4% and the specificity was 88.5% +/- 12.5%. The sensitivity for the control rats with normal paws was 82.6% +/- 14.9% and we did not assess specificity of the normal paws.

### Safety studies based on histology

**Figure 4:**
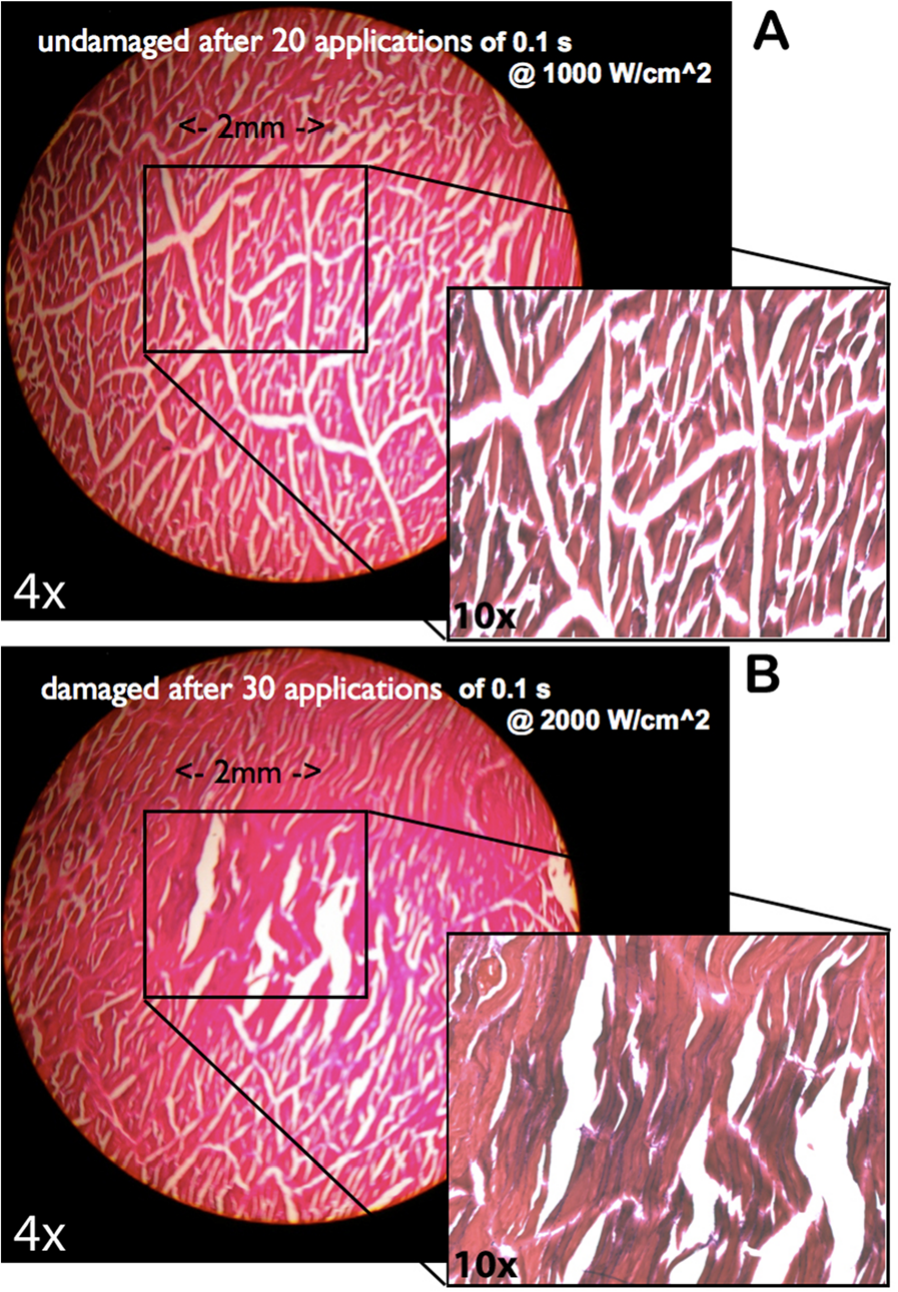
**Histology. A**. Sectioned muscle after 20 applications of 0.1 s iFU pulses at an intensity of 1000 W/cm^2^ and dose of 2000 (W*s)/cm^2^) per pulse. The tissue was undamaged after this application. Large image is 4X magnification, inset is 10X. **B**. Sectioned muscle after 30 applications of 0.1 s iFU pulses at an intensity of 2000 W/cm^2^ and net dose of 6000 (W*s)/cm^2^). This tissue sample shows damage. Large image is 4X magnification, inset is 10X.

Twenty separate 0.1 s applications of iFU spaced ten seconds apart at an intensity of 1000 W/cm^2^ produced no observable cellular damage in tissue taken from seven animals (Figure 4A). In contrast, 30 separate applications of iFU spaced ten seconds apart, each with a duration 0.1 s at an intensity of 2000 W/cm^2^ did produce observable damage acutely in 4 animals (Figure 4B), observed at the focus: 4 mm below the surface of the tissue encompassing an area of tissue measuring 1–2 mm in diameter.

### Safety studies based on theoretical concerns

We calculated the threshold for potential thermal damage in order to add another measure for the safety of our protocol. We used muscle as the model tissue, as this is most representative of the tissue that we believe the device stimulated. We found that over 40% of our tests yielded threshold results above the dose estimated to cause thermal damage. Table 3 summarizes these results. We have also marked the intensity value associated with the theoretical possibility of damage on Figure 3 to give the reader a visual representation of the data.

**Table 3:** Potential damage threshold values by acoustic protocol and device

**Tissue type**	**Muscle**	
**Acoustic protocol**	**0.375 s**	
**Potential damage threshold (W/cm**^**2**^**)**	286.0	
**% of data above t**_**43**_	CFA	Normal*
	41.7%	100%

*note that we tested both CFA-injected animals and normal animals for this duration with the deep device.

## Discussion

We tested the hypothesis that iFU stimulation could assess allodynia associated with subcutaneous painful inflamed tissue. We performed this test by applying iFU to the inflamed, contralateral and normal hind paws of rats, and observed the iFU intensity and dose at which they withdrew their paws from the device in a consistent fashion. We also tested the safety of this procedure by determining the amount of iFU necessary to cause acute damage in muscle relative to our observations of withdrawal behavior by iFU application – a measure of the margin of safety of iFU stimulation. We also calculated estimates of the threshold intensity and dose of iFU necessary to cause damage and compared those to our observation of the amount of iFU necessary to cause a withdrawal from iFU application.

First, we observed that normal rats’ paws and inflamed rats’ paws had significantly different iFU withdrawal thresholds, and those differences correlated well to the standard Hargreaves’ heat lamp test. The correlation of significance between the standard test and the iFU test indicates that iFU threshold for pain or sensation is a reasonable metric for assessing allodynia, important for diagnosing, hence treating chronic pain or other central and/or peripheral nervous system disorders.

We showed functional and histological data consistent with the hypothesis that these iFU protocols do not cause tissue damage. Specifically, the Hargreaves heat withdrawal latencies did not change after iFU application, offering evidence that the rat’s ability to sense thermal stimulation did not change due to iFU stimulation. In addition, we note that Gavrilov, Wright, Dalecki, Dickey, and colleagues applied comparable amounts of ultrasound to themselves and to test subjects without incident [13-16,30]. Moreover, the dose of ultrasound necessary to produce damage to muscle tissue acutely (measured here with histological analysis) was more than 100 times that necessary to cause the observed withdrawal. This preliminary measure of the margin of safety of iFU stimulation is also consistent with published studies of the amount of ultrasound necessary to cause damage in peripheral nerve, a candidate target tissue for the present technology. Specifically, Foley et al. [31] found that an intensity of 7,890 W/cm^2^ and duration 5 seconds [hence a dose of almost 40,000 (W*s)/(cm^2^)] was required to cause acute damage of peripheral nerves. However, we note that all t_43_ calculations (a very conservative means of estimating the potential of thermal damage), when applied to deep-tissue data, suggested that we might have damaged tissue a meaningful percentage of the time, though, again, our direct histological analysis suggests otherwise. We discuss this possibility below.

### Limitations

There are several limitations of our research that motivate future work on this subject: our choice of tissue for the safety studies, our estimates of the temperature induced by iFU during our studies, and the means by which iFU may have stimulated the paws of our rats.

With regard to our safety study, we chose to assess potential acute damage induced by iFU using exposed muscle of Sprague Dawley rats as target tissue, while for the behavioral studies we used the inflamed, contralateral, and normal paws of Fisher rats as target tissue. We chose relatively homogenous muscle tissue as our target for the safety study rather than tissue from the paws of our rats, following the advice of our local histopathologist. Based upon her experience with this kind of tissue she strongly suggested we would find it difficult to identify iFU-induced damage relative to the extensive damage created by the inflammation. In addition, we chose tissue from a different strain of rat in order to conserve the number of rats used for our study. Specifically, the Sprague Dawley rats were from a parallel study and used here for the acute safety study after they had served their purpose in that parallel study. Future work will consider the *long-term* effects of *varying* amounts of repeated stimulation by iFU of tissues *directly relevant* to a given clinical target. Of particular interest may be study of the structure and function of the peripheral nerves in tissue subjected to iFU stimulation. Here, electron microscopy of the peripheral nerve terminals may show subtle alteration of their structure that histopathology would miss.

In addition, we estimated temperature changes induced by iFU during our study through use of the bioheat equation applied to a homogeneous and infinite expanse of tissue. We did so in order to calculate the minimum intensity and dose of iFU that may produce thermal damage. For those calculations we included diffusion but not perfusion, thereby reducing the mathematical complexity, and used reported values of attenuation, a function of absorption plus scattering, more typically measured than absorption alone. These assumptions make our calculations conservative, where they would otherwise reach a higher value were we able to perform simulations that are more realistic. This is of interest because we predicted the possibility of iFU induction of thermal damage to deep, normal tissue for 40% of the time through our use of iFU during some of our experiments, which does not match our histological assay of potential damage, described above. Perhaps one reason for this difference is that we did not constrain the animals to experience the entire iFU application. Indeed, we identified a successful stimulation by iFU by its ability to induce the rat to withdraw its paw. Therefore, they might have withdrawn their paw after successful iFU stimulation before we delivered the entire dose of iFU. Nonetheless, we recognize that the paws of rats as well as clinically relevant tissue in humans are structurally and functionally much more complex than what we have assumed in our metric for thermal damage. Therefore, careful quantification of the actual iFU application time, along with more representative simulations – necessarily much more complex than those considered here – might become warranted as we move closer to human applications of this technology.

The possibility that we have induced cavitation in the paws is worth detailed discussion here. Ultrasound can indeed cavitate tissue. An argument against that occurring here lies in the fact that the dose values we observed to be necessary to cause a paw withdrawal by the rats were mostly under 200 (W*s)/(cm^2^) in uninjured paws. These are substantially smaller than those demonstrated as necessary to create cavitation *in vivo* – approximately 800 (W*s)/(cm^2^), as measured by Hynynen and colleagues [32], albeit at a different frequency. (Of course, the observed threshold for cavitation *in vivo* could only measure cavitation amenable to detection with their device and may therefore have missed cavitation events sufficient to generate sensations.) Nonetheless, we recognize that we inflamed the paws of the rats via injection of Complete Freunds Adjuvant and thereby likely have introduced cavitation nuclei into the tissue. These cavitation nuclei may, in principle, have survived the five to seven days that transpired before we performed our behavioral studies. This possibility seems unlikely to us, however, given the short life of even structured cavitation nuclei (acoustic contrast agents) in blood [33]. Still, we recognize that another explanation for the means by which iFU caused sensations within the paws of our rats is that inflamed paws contain cavitation nuclei while normal paws do not, and that mechanical stimulation by iFU-induced cavitation may have stimulated the rat’s paws. The possibility that cavitation played a role in our studies therefore supports future behavioral studies that simultaneously assess cavitation activity as well as use of degassed CFA.

## Conclusion

There exist magnetic resonance imaging (MRI) guided systems that facilitate the delivery of focused ultrasound deep to the skin [7,9,11]. Given this, our work points to the possibility that iFU – necessarily under image guidance - could serve as a non-invasive method of stimulating hence identifying injured subcutaneous tissue, thereby locating a deep, peripheral anatomical correlate to a patient’s pain. For example, iFU could stimulate individual nerves of amputee patients to create better surgical outcomes, or for implementation of prosthetics. MRI-guided iFU may also help to target tissue for optimal ablation via high intensity focused ultrasound (HIFU) by first locating painful tissue, and may serve as a useful diagnostic technique to guide other treatment modalities. This same method may one day offer a means of assessing allodynia associated with that subcutaneous tissue. Such an approach may also benefit from complementary monitoring of EEG patterns, as has been done for cutaneous stimulation [4]. This idea is consistent with the original work of Gavrilov and colleagues [12,14,15] and suggested by Dalecki et al. [30], and Wright et al. [16], along with our more recent work [17-19]. Given this perspective, successful, definitive testing of image-guided iFU as just described would herald the creation of a new tool for assessing pain associated with subcutaneous anatomical structures, providing useful diagnostic data to guide its treatment, and tracking its treatment through time.

## Competing interests

Drs. Kliot and Mourad have a significant financial interest in PhysioSonics, Inc, one of the sponsors of the research described in this paper.

## Authors’ contributions

The author’s contributions are as follows: experimental design (JG, MK, JL, AM, PM, KP, RS, RT); data collection (JG, AM, KP, RS); data analysis (JG, MG, AM, PM, GT, RT); simulations and calculations (JK, AM, PM); write-up (JG, MG, MK, JK, JL, AM, PM, GT, RT). All authors read and approved the final manuscript.
